# Effect of speed endurance training and reduced training volume on running economy and single muscle fiber adaptations in trained runners

**DOI:** 10.14814/phy2.13601

**Published:** 2018-02-07

**Authors:** Casper Skovgaard, Danny Christiansen, Peter M. Christensen, Nicki W. Almquist, Martin Thomassen, Jens Bangsbo

**Affiliations:** ^1^ Department of Nutrition, Exercise and Sports Section of Integrative Physiology University of Copenhagen Copenhagen Denmark; ^2^ Team Danmark (Danish Elite Sports Organization) Copenhagen Denmark; ^3^ Institute of Sport, Exercise and Active Living (ISEAL) Victoria University Melbourne Australia

**Keywords:** Intense training, muscle fiber type‐specific adaptations, muscular adaptations, sprint interval training

## Abstract

The aim of the present study was to examine whether improved running economy with a period of speed endurance training and reduced training volume could be related to adaptations in specific muscle fibers. Twenty trained male (*n* = 14) and female (*n* = 6) runners (maximum oxygen consumption (VO_2_‐max): 56.4 ± 4.6 mL/min/kg) completed a 40‐day intervention with 10 sessions of speed endurance training (5–10 × 30‐sec maximal running) and a reduced (36%) volume of training. Before and after the intervention, a muscle biopsy was obtained at rest, and an incremental running test to exhaustion was performed. In addition, running at 60% vVO_2_‐max, and a 10‐km run was performed in a normal and a muscle slow twitch (ST) glycogen‐depleted condition. After compared to before the intervention, expression of mitochondrial uncoupling protein 3 (UCP3) was lower (*P *< 0.05) and dystrophin was higher (*P* < 0.05) in ST muscle fibers, and sarcoplasmic reticulum calcium ATPase 1 (SERCA1) was lower (*P* < 0.05) in fast twitch muscle fibers. Running economy at 60% vVO_2_‐max (11.6 ± 0.2 km/h) and at v10‐km (13.7 ± 0.3 km/h) was ~2% better (*P* < 0.05) after the intervention in the normal condition, but unchanged in the ST glycogen‐depleted condition. Ten kilometer performance was improved (*P* < 0.01) by 3.2% (43.7 ± 1.0 vs. 45.2 ± 1.2 min) and 3.9% (45.8 ± 1.2 vs. 47.7 ± 1.3 min) in the normal and the ST glycogen‐depleted condition, respectively. VO_2_‐max was the same, but vVO_2_‐max was 2.0% higher (*P* < 0.05; 19.3 ± 0.3 vs. 18.9 ± 0.3 km/h) after than before the intervention. Thus, improved running economy with intense training may be related to changes in expression of proteins linked to energy consuming processes in primarily ST muscle fibers.

## Introduction

Speed endurance training (SET; 10–40 sec repeated “all‐out” efforts with rest periods lasting >5 times the exercise bouts) with a concomitant reduced training volume has been found to improve endurance performance in association with better running economy at submaximal speeds in trained runners (Bangsbo et al. 2009; Bangsbo 2015). However, the mechanisms causing the improved running economy are not clearly identified, but may be related to metabolic changes in the trained muscles (Saunders et al. 2004).

Training‐induced improvement in running economy may be due to higher mitochondrial efficiency, that is, higher ATP/O2, which could be due to reduced uncoupled respiration. The mitochondrial uncoupling protein 3 (UCP3) is suggested to be involved in thermogenesis by dispersing energy as heat instead of converting it to ATP (Gong et al. 1997; Boss et al. 2000) and improved running economy may therefore be related to reduced levels of muscle UCP3. In agreement, cross‐sectional studies have shown that endurance‐trained subjects have lower muscle UCP3 expression and better running economy than untrained subjects (Russell et al. 2003a,b; Mogensen et al. 2006). However, Iaia et al. (2009) found no change in whole muscle UCP3 level although running economy improved after 4 weeks of SET and a 65% reduced training volume. Thus, studies should investigate whether changes in the single muscle fiber expression of UCP3 could be related to changes in running economy.

The transfer of muscle force produced by the actomyosins involves a secondary matrix of proteins that transmit the muscle force along and between muscle fibers and out to the extracellular matrix. Cytoskeleton proteins, such as dystrophin, have been identified as playing a role in this muscle force transmission (Rybakova et al. 2000; Prins et al. 2009) and changes in the expression of these proteins could influence the integrity and the strength of the muscle (Hughes et al. 2015). Hence, increased expression of muscle dystrophin may result in increased rate of force development, increased muscular power output and greater storage and return of elastic energy thereby lowering the cost of running (i.e., improve running economy).

Another potential cause of training‐induced improvements in running economy is lowered muscle expression of the sarcoplasmic reticulum (SR) Ca^2+^‐ATPase (SERCA) pumps, as they are suggested to be responsible for up to 50% of the ATP used during muscle activity (Clausen et al. 1991; Walsh et al. 2006; Smith et al. 2013). Studies have shown that speed endurance training modulates skeletal muscle fiber type distribution in soccer players (Gunnarsson et al. 2012) and runners (Skovgaard et al. 2014), which has been found together with lowered SERCA1 expression (Skovgaard et al. 2014) and improved running economy. Muscle fibers with high SERCA1 expression have a faster release and uptake of Ca^2+^ (Delbono and Meissner 1996; Froemming et al. 2000) and lowered expression of SERCA1 may therefore reduce the energy turnover during exercise.

An increase in the respiratory capacity of skeletal muscle permits the use of less oxygen per mitochondrial respiratory chain for a given submaximal running speed (Saunders et al. 2004). Slow twitch (ST) muscle fibers have higher mitochondrial content and are more dependent on oxidative metabolism than fast twitch (FT) muscle fibers (Berchtold et al. 2000; Schiaffino and Reggiani 2011). However, Jansson and Kaijser (1977) reported that, unlike a control group of varying physical fitness, there was no difference in succinate dehydrogenase muscle activity between ST and FT fibers in gastrocnemius muscle of elite orienteers, suggesting that FT fibers have the ability to metabolically adapt to high oxidative demands (Jansson and Kaijser 1977). Metabolic adaptations in FT fibers may therefore contribute to improving running economy after intense training, such as SET, targeting both ST and FT fibers (Egan and Zierath 2013). In support, augmented mRNA response related to mitochondrial biogenesis (peroxisome proliferator‐activated receptor‐*γ* coactivator‐1, PGC‐1*α*) and metabolism (hexokinase II and pyruvate dehydrogenase kinase‐4, PDK4) in trained subjects was observed following a SET session (Skovgaard et al. 2016). Furthermore, PGC‐1a mRNA has been shown to increase in an exercise intensity‐dependent manner (Egan et al. 2010; Nordsborg et al. 2010). Regular intense training may therefore lead to higher oxidative capacity, possibly due to oxidative adaptations in FT fibers, which in turn could contribute to the improved running economy as a result of the intense training (Iaia et al. 2008; Bangsbo et al. 2009; Iaia and Bangsbo 2010; Skovgaard et al. 2014).

In vitro studies have shown that the energy cost of contraction is higher in FT than ST fibers (Crow and Kushmerick 1982; Barclay et al. 1993; He et al. 2000). This was confirmed in vivo by Krustrup et al. (2008) who observed that the oxygen uptake for at given exercise intensity was higher when ST fibers were blocked by a neuromuscular blocking agent. And reports by Krustrup et al. (2004), who depleted the ST fibers the day before submaximal exercise, that the glycogen depletion of ST fibers enhanced the recruitment of FT fibers and elevated the energy requirement by 7% (Krustrup et al. 2004). By using the approach, of depleting ST fibers the day before exercise (Krustrup et al. 2004), before and after a SET period, it may be possible to study whether a change in running economy is caused by specific adaptations in FT fibers.

Thus, the aims of the present study were in trained runners to investigate the effect of intensified training, in the form of speed endurance training and a reduced volume of aerobic training, on running economy and adaptation of single muscle fibers. We hypothesized that FT muscle fibers would adapt to the training by lowered expression of UCP3 and SERCA1, and increased expression of dystrophin and CS, which would be associated with improved running economy and 10‐km running performance.

## Methods

### Subjects

Twenty‐six trained runners commenced the study. Six subjects did not complete the intervention period due to personal circumstances (*n* = 4) or low adherence to the training program (*n* = 2). Thus, a total of twenty trained male (*n* = 14) and female (*n* = 6) runners with an average age, height, body mass, and maximum oxygen consumption (VO_2_‐max) of 28.1 ± 4.5 years, 177.5 ± 9.9 cm, 72.5 ± 10.6 kg, and 56.4 ± 4.6 mL/min/kg, respectively, (males: 28.8 ± 4.8 years, 181.8 ± 7.9 cm, 77.8 ± 6.6 kg, 58.1 ± 3.4 mL/min/kg; females: 27.4 ± 3.7 years, 169.0 ± 5.6 cm, 59.9 ± 6.9 kg, 52.5 ± 4.9 mL/min/kg; means ± SD), completed the study. After receiving written and oral information about the study and the possible risks and discomforts associated with the experimental procedures, all subjects gave their written informed consent to participate. The study conformed to the Code of Ethics of the World Medical Association (Declaration of Helsinki) and was approved by the Ethics Committee of the capital region of Copenhagen (Region Hovedstaden).

### Design

**Figure 1:**
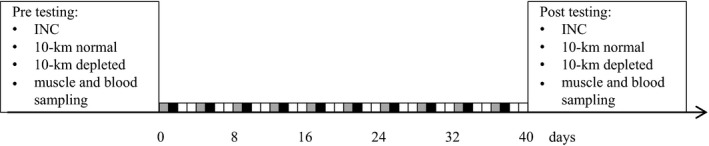
Testing before (Pre) and after (Post) 5 blocks/40 days of speed endurance training and reduced training volume in trained runners. Small grey, black and white boxes on the timeline are days with aerobic moderate‐intensity training, speed endurance training and rest days, respectively. INC: incremental test to exhaustion.

The study lasted 40 days and consisted of 10 sessions of supervised speed endurance training (SET) and 10 sessions of aerobic moderate‐intensity (AM) training (Fig. 1). Total running distance during the intervention period was reduced (*P* < 0.05) by 36% compared to before the intervention (mean ± SE, 16 ± 1 vs. 25 ± 2 km/week).

### Screening and familiarization

Before being included in the study, subjects performed a 10‐km running test and an incremental treadmill test to exhaustion with pulmonary VO_2_ measured by a breath‐by‐breath gas analyzing system (Oxycon Pro; Viasys Healthcare, Hoechberg, Germany), and heart rate (Polar Team^2^ transmitter; Polar Electro Oy, Kempele, Finland) collected throughout the test.

### Training

SET was performed on day two and six of an 8‐day cycle at Østerbro Stadium, Copenhagen, on an outdoor 400‐m running track. In first and final SET session, subjects completed six bouts of 30‐sec running. The first bout was performed with near‐maximal intensity, whereas the remaining five bouts were performed with maximal intensity and distance covered was measured. For the remaining eight SET sessions, subjects completed ten bouts of 30‐sec “all‐out” running. In all sessions, running bouts were separated by 3.5 min of recovery (walking ~200 m to the start‐line). SET sessions were supervised, but the subjects performed the SET sessions on their own, if they were unable to participate in the supervised training (85 ± 4% adherence to the supervised SET).

AM training was performed on the first and fifth day during the 8‐day cycle. These sessions were not supervised, but subjects kept a training log to record exercise distance, time and intensity. A Polar FT7 (Polar Electro Oy, Kempele, Finland) or personal watch with HR monitor was used to record exercise intensity and training logs was continuously analyzed. The adherence to the AM training sessions was 93 ± 3% with a weekly duration of 68 ± 5 min and with an average heart rate of 83 ± 1% of HR_max_.

### Testing

Tests were performed on separate days interspersed by at least 48 hours, on the same treadmill in the Exercise Physiology laboratory at August Krogh Institute, Department of Nutrition, Exercise and Sports, University of Copenhagen, before and after the intervention. Tests included: (1) an incremental running test to exhaustion (INC); (2) repeated bouts of 6‐min submaximal running followed by a 10‐km running test on a running track in a normal condition; (3) repeated bouts of 6‐min submaximal running followed by a 10‐km running test on a running track in a ST glycogen‐depleted condition; (4) a muscle biopsy and a blood sample collected at rest after an overnight fast (Fig. 1).

All tests were carried out at the same time of day. Subjects refrained from strenuous physical activity, alcohol and caffeine 24 h before testing. Subjects were instructed to keep a diary journal 2 days before and during the first series of tests, and to replicate this diet when tested again.

#### The incremental running test to exhaustion

INC consisted of 2 min of walking at 5 km/h, 6 min at the subject's individual average 10‐km running pace determined at the 10‐km screening test before the intervention (v10 km; 13.7 ± 0.3 km/h), and 2 min at 14 or 15 km/h (dependent on v10 km), after which the speed increased by 1 km/h every minute until exhaustion. During INC, VO_2_‐max, defined as the highest average value achieved over a 30‐sec period (Howley et al. 1995), and maximal incremental speed (vVO_2_‐max) {[vVO_2_‐max = *V* ^f^ + (*T* ^i^/60)], where *V* ^f^ is the final velocity obtained and *T* ^i^ is the time spent at the final speed level} were determined. Attaining of maximal heart rate (HR) (judged against the screening test) and an RER value of >1.15 were used as criterions. During the last part of the test, the subjects were verbally encouraged to continue their effort until voluntary termination of the test. Before the test, body mass was measured and subjects wore a Polar Team^2^ HR monitor around their chest for continuous HR recordings. Pulmonary VO_2_ was measured by use of Oxycon Pro, which was calibrated prior to each test.

#### Muscle and blood sampling

Sampling of muscle and blood was performed between 7 and 11 am after an overnight fast. Using the Bergström procedure (Bergstrom 1962), a muscle biopsy was collected with a 5‐mm needle from a standardized depth of 5 cm in the middle of m. vastus lateralis of the right leg at rest using local anesthesia (1 mL; 20 mg/L lidocaine without adrenaline). The muscle sample (~100 mg wet weight) was immediately frozen in liquid N_2_ and stored at −80°C until further analysis. Next, a catheter was inserted in the antecubital vein, and a ~7‐mL blood sample was collected and stored on ice until being analyzed.

#### 10‐km running tests

Both before and after the intervention, two 10‐km running tests were performed on a 400‐m outdoor running track (Østerbro Stadium, Copenhagen) under similar weather conditions (~20°C, partly cloudy, light winds) between the beginning of July and end of August. The 10‐km tests were conducted in a randomized order either without (normal) or after a muscle ST glycogen depletion protocol that was performed the day before the test (see later). Each 10‐km running test was preceded by two bouts of 6 min of running, separated by 20 min of rest, on a treadmill at the subject's individual 60% vVO_2_‐max (11.6 ± 0.2 km/h) with respiratory and HR measurements. After these bouts, subjects biked to Østerbro Stadium (1‐km, slow pace) for the 10‐km test.

#### Muscle slow‐twitch glycogen depletion protocol

The protocol was based on the findings from the study by Krustrup et al. (2004) who used a 3‐h cycling protocol (~50% VO_2_‐max) to deplete ST fibers the day before 20‐min of submaximal cycling. The authors reported that the glycogen depletion of ST fibers (51 and 44% of the ST fibers were empty and almost empty of glycogen, respectively, and less than 2% of the FT fibers were empty of glycogen) enhanced the recruitment of FT fibers (Krustrup et al. 2004). The protocol is verified by previous findings that ST fibers are exclusively active at 50% VO_2_‐max when subjects have normal muscle glycogen levels (Gollnick et al. 1974; Vøllestad and Blom 1985).

The subjects completed a 3‐h exercise protocol consisting of 60 min of cross‐training, 30 min of cycling, 30 min of running, and 60 min of striding at a low speed to deplete glycogen in ST muscle fibers of the calves and thigh muscles. The protocol was chosen to minimize muscle soreness from eccentric contractions while mimicking the movement pattern of running. During the protocol, subjects’ HR was monitored to ensure they exercised at 60–65% of HR_max_ (~50% VO_2_‐max). Average HR during the 3‐h depletion protocol was the same before and after the intervention (120 ± 1 vs. 120 ± 1 bpm; 63 ± 0 vs. 63 ± 0% HR_max_). The protocol started at 6:30 pm and finished around 10:00 pm and subjects were allowed water ad libitum. After termination of the protocol, subjects were given a diet consisting of 5E% carbohydrate, 35E% protein, and 60E% fat with a total energy intake of 30 kJ/kg body mass at dinner and 10 kJ/kg at breakfast. Breakfast was consumed 2 h before the 10‐km running test, which started at 8:00 am.

#### Whole muscle protein expression

Western blotting was performed to determine protein expression as described previously (Skovgaard et al. 2014). In short, ~2.5 mg dry weight (dw; freeze‐dried for a minimum of 24 h) of each muscle sample was dissected free from blood, fat, and connective tissue. Samples were homogenized for 1 min at 28.5 Hz (Qiagen Tissuelyser II; Retsch) in a fresh batch of ice‐cold buffer containing (in mM) 10% glycerol, 20 Na‐pyrophosphate, 150 NaCl, 50 HEPES (pH 7.5), 1% NP‐40, 20 *β*‐glycerophosphate, 2 Na_3_VO_4_, 10 NaF, 2 PMSF, 1 EDTA (pH 8), 1 EGTA (pH 8), 10 *μ*g/mL aprotinin, 10 *μ*g/mL leupeptin, and 3 benzamidine, after which they rotated for 1 h at 4°C, and centrifuged at 18,320*g* for 20 min at 4°C to exclude nondissolved structures. The supernatant (lysate) was collected and used for further analysis. Total protein concentration in each sample was determined by a BSA standard kit (Thermo Scientific), and samples were mixed with 6× Laemmli buffer (7 mL 0.5 mol/L Tris‐base, 3 mL glycerol, 0.93 g DTT, 1 g SDS, and 1.2 mg bromophenol blue) and ddH_2_O to reach equal protein concentration before protein expression was determined by western blotting.

Equal amounts of total protein (6–12 *μ*g depending on the protein of interest) were loaded in each well of precast gels (Millipore). All samples from each subject were loaded on the same gel. Proteins were separated according to their molecular weight by SDS‐PAGE and semi‐dry transferred to a 0.45 *μ*m PVDF membrane (Bio‐Rad). The membranes were blocked in either 2% skimmed milk or 3% BSA in TBST, including 0.1% Tween‐20 before an overnight incubation with rocking in primary antibody at 4°C. The primary antibodies used were: (ab. cat number and company, respectively): sarcoplasmic reticulum Ca^2+^‐ATPase 1 (SERCA1; MA3‐912; Thermo Scientific), sarcoplasmic reticulum Ca^2+^‐ATPase 2 (SERCA2; N‐19 Sc‐8095; Santa Cruz Technology), actin (A2066; Sigma Aldrich), mitochondrial uncoupling protein 3 (UCP3; AB3046; Millipore). The membranes were then incubated for 1 h at room temperature in horseradish peroxidase conjugated secondary antibody (rabbit anti‐sheep (P‐0163, DAKO), rabbit anti‐goat (P‐0449, DAKO), goat anti‐mouse (P‐0447, DAKO) or goat anti‐rabbit IgM/IgG (4010‐05; Southern Biotech), depending on the primary antibody source).

The protein bands were visualized with ECL (Millipore) and recorded with a digital camera (ChemiDoc MP Imaging System, Bio‐Rad Laboratories). For each muscle sample, protein expression was determined in duplicate on individual gels. Quantification of the band intensity was performed using Image Lab version 4.0 (Bio‐Rad Laboratories). Each band was normalized to two control samples of human, whole‐muscle homogenate that were loaded onto every gel.

#### Single muscle fiber protein expression

To determine the protein expression of citrate synthase (CS), UCP3 as well as SERCA‐ and myosin heavy chain (MHC) isoforms in different muscle fiber types, 88 ± 5 single‐fiber segments were collected from each freeze‐dried muscle biopsy. Individual segments were isolated under a microscope at room temperature using fine jeweler's forceps, and were individually incubated for 1 h at room temperature in microfuge tubes containing 10 *μ*L of denaturing buffer (0.125 mol/L Tris‐HCl, 10% glycerol, 4% SDS, 4 mol/L urea, 10% mercaptoethanol, and 0.001% bromophenol blue, pH 6.8) (Murphy, 2011). The denatured segments were stored at −80°C until being analyzed for fiber type and grouped accordingly as described below.

The fiber type of fiber segments was determined using dot blotting. 1.5 *μ*L of each denatured sample was spotted onto two PVDF membranes, which were pre‐activated in 95% ethanol and pre‐equilibrated in transfer buffer (25 mmol/L Tris, 192 mmol/L glycine, pH 8.3, 20% methanol). After drying completely at room temperature, the membranes containing samples were reactivated in ethanol and re‐equilibrated in transfer buffer, before being blocked in 5% skim milk in TBST for 5–30 min. One membrane was then incubated by gentle rocking with MHCI antibody (1:200 in 1% BSA with PBST; mouse monoclonal IgM, clone A4.840, Developmental Studies Hybridoma Bank (DSHB)), and the other with MHCIIa antibody (mouse monoclonal IgG, clone A4.74, DSHB) for 2 h at room temperature. After a quick wash in TBST, secondary antibody was applied (1:10,000), and protein signals quantified as described under *Whole muscle protein expression* (section above).

The remaining part of each denatured fiber segment (7 *μ*L) was pooled into groups of ST or FTa fibers depending on MHC expression. The number of segments entailed in each pool of fibers per biopsy was 15 ± 2 (range: 8–42) for ST and 18 ± 2 (range 8–39) for FTa fibers before the intervention, and 19 ± 3 (range: 7–55) and 18 ± 2 (range 7–41), respectively, after the intervention. Hybrid fibers (expressing multiple MHC isoforms) were excluded from analysis. Protein expression was determined in pools of ST and FTa fibers using western blotting as detailed in the section above. The primary antibodies used were: (ab. cat number and company, respectively): CS (ab96600, Abcam), UCP3 (AB3046; Millipore) SERCA1 (MA3‐912; Thermo Scientific), SERCA2 (N‐19 Sc‐8095; Santa Cruz Technology). Pools of fibers from biopsies obtained before and after the intervention was loaded on the same gel (stain‐free, 4–15%, precast), along with either a calibration curve or two loading controls of whole‐muscle homogenate. Protein bands were quantified by normalizing each band to the total protein content in each lane on the stain‐free gel.

#### Muscle enzyme activity

Muscle enzyme activity was determined by use of ~2.5 mg dw muscle tissue dissected free from blood, fat, and connective tissue, which was homogenized (1:400) in a 0.3 mol/L phosphate buffer (pH 7.7) by 2 rounds of 30‐sec using a TissueLyser II (Retch, Germany). Maximal activity of CS, *β*‐hydroxyacyl‐CoA‐dehydrogenase (HAD) and phosphofructokinase (PFK) was determined fluorometrically with NAD‐NADH coupled reactions (Lowry and Passonneau 1972) on a Fluoroskan Ascent apparatus (Thermo Scientific) using Ascent Software version 2.6.

#### Blood analysis

A total of ~7 mL blood was drawn in a heparinized 2‐mL syringe and a 5‐mL syringe at rest. A part of the 2‐mL blood sample (~1.5 mL) and the 5‐mL sample (split into 2 × 2 mL tubes containing 30 *μ*L EDTA) were centrifuged at 20,000 g for ~2 min and the remaining whole blood from the 2‐mL sample (~0.5 mL) was stored on ice for further analyses. After centrifugation, the plasma was transferred into tubes that were placed in ice‐cold water until they were stored at −20°C. Plasma samples were subsequently analyzed for testosterone and cortisol, creatine kinase (CK) and immunoglobulin A (IgA). CK activity was analyzed by enzymatic kinetic assay methods (Roche Diagnostic, Mannheim, Germany) using a Hitachi 912 (Roche Diagnostic, Indianapolis). IgA was determined using an immunoturbidimetric assay method (Horiba, Montpellier, France) on an automatic analyzer (Pentra C400, Horiba, Montpellier, France). Testosterone and cortisol was determined using ELISA kits (R&D Systems, Inc. Minneapolis). Whole blood was analyzed for hemoglobin, hematocrit and HCO_3_ at rest (ABL800 Flex; Radiometer Medical, Copenhagen, Denmark).

#### Running economy

Running Economy (RE) was calculated using the following formula:

$$\text{RE}\left(\text{mL}O_{2}/\text{kg}/\text{km}\right)={\text{VO}}_{2}\left(\text{mL/min}\right)\cdot 60\;\text{min/h}/\text{BM (kg)}\cdot \text{running speed}\left(\text{km/h}\right)$$

where VO_2_ is the average value during the last 2 min of running for the two intervals at 60% vVO_2_‐max and v10‐km, and BM is body mass.

### Statistics

Paired *t* tests were used to evaluate the effect of the intervention (Pre vs. Post) with two‐way ANOVA repeated measures being used to evaluate the effect of glycogen condition (normal vs. ST glycogen‐depleted) on 10‐km running performance and running economy (at 60% vVO_2_‐max). Level of significance was set at *P* < 0.05. A Student‐Newman Keuls post‐hoc test was applied in case significance was reached in the ANOVA. Absolute data values was used and presented as means ± SE unless otherwise stated.

## Results

### Pulmonary oxygen uptake and heart rate during submaximal exercise

**Figure 2:**
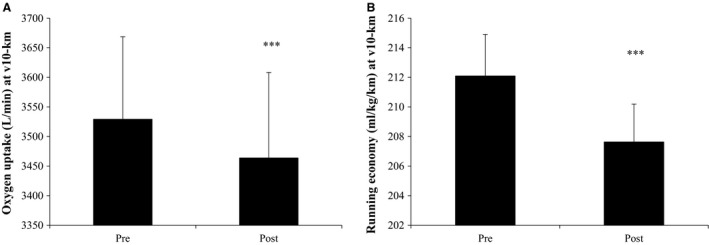
Oxygen uptake (A) and running economy (B) at v10‐km before (Pre) and after (Post) 5 blocks/40 days of speed endurance training and reduced training volume in trained runners. Values are means ± SE. ***Post different (*P* < 0.001) to Pre.

Pulmonary VO_2_ during running at v10‐km was 1.9% lower (*P* < 0.05) after compared to before the intervention (3.46 ± 0.14 vs. 3.53 ± 0.14 L/min), and running economy was improved by 2.1% (*P* < 0.05; 207.6 ± 2.6 vs. 212.1 ± 2.8 mL/kg/km) (Fig. 2). Mean HR at v10‐km was 1.7% lower (*P* < 0.05) after than before the intervention (162 ± 2 vs. 165 ± 2 bpm).

**Figure 3:**
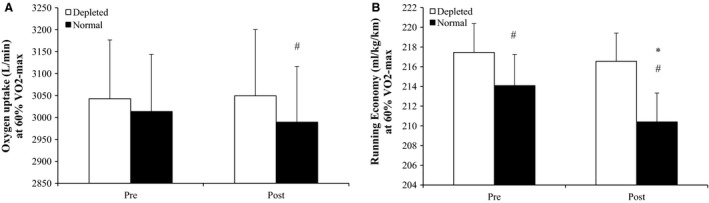
Oxygen uptake (A) and running economy (B) at 60% VO2‐max in depleted and normal conditions before (Pre) and after (Post) 5 blocks/40 days of speed endurance training and reduced training volume in trained runners. Values are means ± SE. *Post different (*P* < 0.05) to Pre; ^#^difference (*P* < 0.05) within time‐point.

In the normal condition, pulmonary VO_2_ at 60% vVO_2_‐max was the same before and after the intervention (3.01 ± 0.13 vs. 2.99 ± 0.13 L/min), whereas running economy was 1.7% better (*P* < 0.05) after compared to before the intervention (210.4 ± 2.9 vs. 214.1 ± 3.2 mL/kg/km) (Fig. 3). In the ST glycogen‐depleted condition, pulmonary VO_2_ at 60% vVO_2_‐max (3.05 ± 0.15 (Post) vs. 3.04 ± 0.13 (Pre) L/min) and running economy (216.5 ± 2.9 (Post) vs. 217.4 ± 2.9 (Pre) mL/kg/km) did not change with the intervention (Fig. 3).

Before the intervention, pulmonary VO_2_ at 60% vVO_2_‐max was the same in normal and ST glycogen‐depleted condition, whereas after the intervention, pulmonary VO_2_ was 2.0% lower (*P* < 0.01) in normal than ST glycogen‐depleted condition. Before and after the intervention, running economy was 1.6% and 2.9% better (*P* < 0.05), respectively, in the normal compared to the ST glycogen‐depleted condition (Fig. 3).

HR during running at 60% vVO_2_‐max in normal and ST glycogen‐depleted condition did not change with the intervention, and there were no differences between conditions.

### Expression of proteins in muscle homogenate

**Figure 4:**
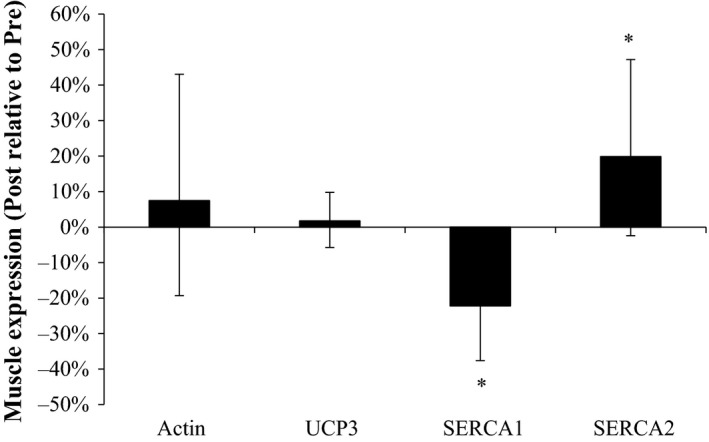
Protein expression of actin; UCP3, mitochondrial uncoupling protein 3; SERCA1 and 2, sarcoplasmic reticulum calcium ATPase, before (Pre) and after (Post) 5 blocks/40 days of speed endurance training and reduced training volume in trained runners. Values are geometric means ± 95% confidence interval (CI) (Post relative to Pre). *Post different (*P* < 0.05) from Pre.

Expression of SERCA2 in muscle homogenate was 20% higher (*P* < 0.05) after compared to before the intervention, whereas expression of muscle SERCA1 was 22% lower (*P* < 0.05). Expression of muscle actin and UCP3 did not change with the intervention (Fig. 4).

### Expression of proteins in single muscle fibers

**Figure 5:**
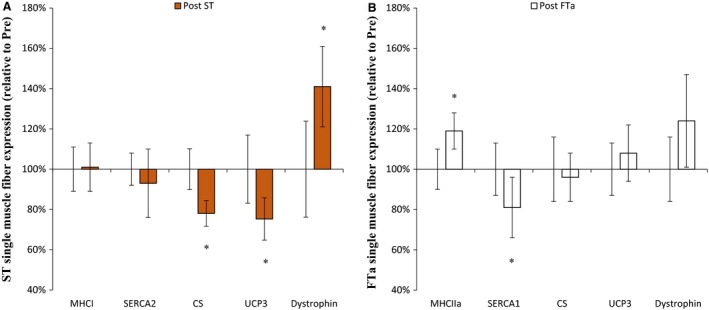
ST (A) and FTa (B) single muscle fiber expression of MHCI and II, myosin heavy chain; SERCA1 and 2, sarcoplasmic reticulum calcium ATPase; CS, citrate synthase; UCP3, mitochondrial uncoupling protein 3; and dystrophin, before (Pre) and after (Post) 5 blocks/40 days of speed endurance training and reduced training volume in trained runners. Values are means ± SE (relative to Pre). *Post different (*P* < 0.05) from Pre.

After compared to before the intervention, expression of muscle CS and UCP3 in ST fibers was 22% and 25%, respectively, lower (*P* < 0.05), and expression of muscle dystrophin in ST fibers was 41% higher (*P* < 0.05) (Fig. 5). Expression of muscle SERCA1 was 19% lower (*P* < 0.05) in FTa fibers, and expression of MHCIIa was 19% higher (*P* < 0.05) in FTa fibers after than before the intervention. Expression of SERCA2 and MHCI in the single fiber pools was unchanged with the intervention (Fig. 5).

### Muscle enzymatic activity

Maximal activity of CS, HAD, and PFK was 10.7%, 9.1%, and 23.4%, respectively, higher (*P* < 0.05) after than before the intervention (Table 1).

**Table 1:** Maximal activity of muscle citrate synthase (CS), β‐hydroxyacyl‐CoA‐dehydrogenase (HAD); phosphofructokinase (PFK) at rest before (Pre) and after (Post) 5 blocks/40 days of speed endurance training and reduced training volume in trained runners

	Pre	Post
CS (*μ*mol·g/dw/min)	17.7 ± 2.9	19.6 ± 2.9^a^
HAD (*μ*mol·g/dw/min)	15.6 ± 0.9	17.0 ± 0.7^a^
PFK (*μ*mol·g/dw/min)	72.1 ± 15.3	88.9 ± 13.7^a^

Data are presented as means ± SE.

a Post different (*P* < 0.05) to Pre.

### 10‐km run

**Figure 6:**
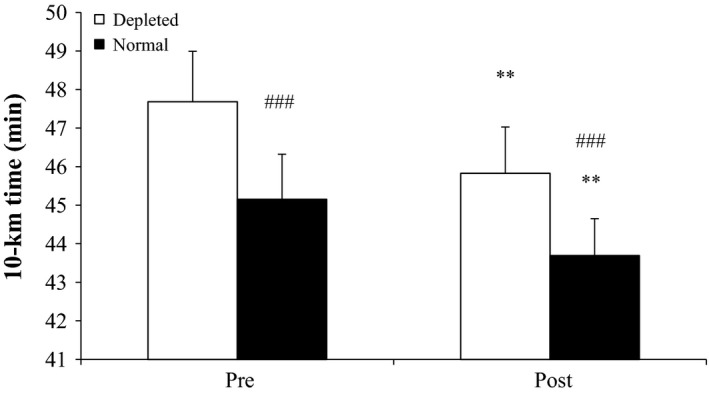
Time to complete a 10‐km run in depleted and normal conditions before (Pre) and after (Post) 5 blocks/40 days of speed endurance training and reduced training volume in trained runners. Values are means ± SE. **Post different (*P* < 0.01) to Pre; ^###^difference (*P* < 0.001) within time‐point.

Compared to before, 10‐km performance in the normal condition improved (*P* < 0.01) by 3.2% (43.7 ± 1.0 vs. 45.2 ± 1.2 min) after the intervention (Fig. 6). In the ST glycogen‐depleted condition, 10‐km performance was 3.9% better (*P* < 0.001) after compared to before the intervention (45.8 ± 1.2 vs. 47.7 ± 1.3 min; Fig. 6). Ten kilometer performance was reduced (*P* < 0.001) to the same degree in the ST glycogen‐depleted compared to the normal condition before (5.3%) and after (4.7%) the intervention (Fig. 6).

### Maximum oxygen uptake, body mass, and heart rate

VO_2_‐max was the same before and after the intervention (4.06 ± 0.16 vs. 4.13 ± 0.18 L/min; 56.4 ± 1.0 vs. 56.3 ± 1.2 mL/min/kg), but vVO_2_‐max was 2.0% higher (*P* < 0.05) after compared to before (19.3 ± 0.3 vs. 18.9 ± 0.3 km/h). Peak heart rate during INC was the same before and after the intervention (187 ± 2 vs. 188 ± 2 bpm) as well as body mass (72.5 ± 2.4 vs. 72.9 ± 2.3).

### Blood variables

Blood hematocrit and concentration of hemoglobin as well as plasma concentrations of testosterone, cortisol, CK and HCO_3_^−^ were the same before and after the intervention. Compared to before the intervention, testosterone to cortisol ratio was 31.3% higher (*P* < 0.05) and plasma IgA level was 4.0% higher (*P* < 0.05) after (Table 2).

**Table 2:** Plasma testosterone (T), cortisol (C) and T:C ratio, hemoglobin and hematocrit, creatine kinase, (CK), and Immunoglobulin A, (IgA) and HCO_3_^−^ before (Pre) and after (Post) 5 blocks/40 days of speed endurance training and reduced training volume in trained runners

	Pre	Post
Testosterone (nmol/L)	24.2 ± 2.9	25.4 ± 3.5
Cortisol (nmol/L)	195.8 ± 21.2	168.3 ± 25.2
*t*:*c* ratio	0.16 ± 0.03	0.21 ± 0.03^a^
Hemoglobin (mmol/L)	8.9 ± 0.2	8.5 ± 0.2
Hematocrit (%)	44.2 ± 1.1	41.8 ± 0.9
CK (U/L)	208 ± 41	155 ± 17
IgA (g/L)	1.98 ± 0.19	2.06 ± 0.19^a^
HCO_3_^−^ (mmol/L)	24.3 ± 0.4	25.0 ± 0.7

Data are presented as means ± SE.

a Post different (*P* < 0.05) to Pre.

## Discussion

The main findings of the present study were that a period of intense and reduced volume of training in trained runners improved running economy together with higher expression of dystrophin and lowered expression of UCP3 in ST muscle fibers as well as lower expression of SERCA1 in FTa muscle fibers. In addition, compared to the normal condition, 10‐km running performance and running economy was equally reduced after the ST muscle glycogen‐depletion protocol before and after the intervention period.

The better running economy at 60% vVO_2_‐max and v10‐km after the intervention period is in accordance with findings in other studies of intense training and lowered training volume in trained runners (Bangsbo et al. 2009; Iaia and Bangsbo 2010; Skovgaard et al. 2014). In the ST glycogen‐depleted condition, where a higher recruitment of FT fibers would be expected, the running economy remained unchanged with training, suggesting that it was mainly changes in ST fibers that caused the improvement in running economy in the normal condition. In accordance, the expression of UCP3 in ST fibers was lowered by training in the present study. As mechanical energy efficiency is negatively related to UCP3 expression (Russell et al. 2003a,b; Mogensen et al. 2006), this suggests that the reduced UCP3 expression in ST fibers may have improved the mitochondrial efficiency, and thereby running economy. On the other hand, reduced energy expenditure during submaximal exercise was reported in a study where trained subjects (VO_2_‐max: 56 ± 1 mL/min/kg) performed 4 weeks of speed endurance training (8–12 × 30‐sec at maximum speed; 3 times/week) with a 65% reduced training volume without change in the expression of whole muscle UCP3 (Iaia et al. 2009). It could be speculated that the reduction in training volume was too large to elicit changes in UCP3 expression or that a potential reduced expression of UCP3 in ST fibers was undetected by the analysis of whole muscle tissue (Iaia et al. 2009).

In addition, the expression of dystrophin, a protein that connects the sarcomere and the extracellular matrix (Hughes et al. 2015), increased in ST muscle fibers with the training intervention. An important function of dystrophin is to transmit forces generated by the actin‐myosin cross‐bridge (Chopard et al. 2005), and the higher expression of dystrophin in ST fibers may have enhanced the structural integrity of the ST muscle fibers and thereby influenced running economy.

The intervention period also led to lowered expression of SERCA1 in FT muscle fibers, which has also been found in studies of endurance training (Majerczak et al. 2008, 2012; Green et al. 2011). The lower expression of muscle SERCA1 may have reduced the energy turnover during exercise, since calcium handling by the ATP dependent SERCA pumps is reported to be responsible for up to 50% of total energy usage (Clausen et al. 1991; Walsh et al. 2006; Smith et al. 2013), and, thus, may have contributed to the better running economy after the intervention period.

The finding of improved 10‐km running performance after the intervention period is in agreement with other studies investigating the effect of intense training and lowered training volume in trained runners (Bangsbo et al. 2009; Iaia and Bangsbo 2010; Skovgaard et al. 2014). The novel finding in the present study was that the magnitude of the difference between 10‐km running in normal versus ST fiber glycogen‐depleted condition was the same before and after the training period. This observation suggests that any effect of the intervention on the oxidative capacity of the FT fibers was small, which is supported by the finding that the expression of CS in the FT fibers did not change with the intervention. In agreement, a 7‐week intense training period (12 × 30‐sec maximal sprints 2.5 times/week and 5 × 4‐min intervals (at a heart rate (HR) of 89% HR_max_) 1.5 times/week) with a 50% reduction in training volume, did not change expression of muscle CS and COX‐4 in segments of FT fibers in well trained cyclists (VO_2_‐max: 59 ± 4 mL/min/kg) (Christensen et al. 2015). Collectively, these findings suggest that intense training with a decrease (36–50%) in training volume does not affect oxidative proteins in FT muscle fibers in trained subjects. Nevertheless, the mixed muscle CS activity was elevated with the intervention and may have contributed to the better 10‐km performance.

In agreement with other studies on the effect of speed endurance training and reduced training volume in runners (Bickham et al. 2006; Iaia et al. 2008; Bangsbo et al. 2009; Iaia and Bangsbo 2010; Skovgaard et al. 2014), VO_2_‐max did not change with the intervention and cannot explain the improved 10‐km performance. Based on the performance during the 10‐km run, VO_2_‐max and running economy, the fraction of FVO_2_‐max [FVO_2_‐max = 10‐km velocity (km/hr)*running economy at v10‐km (mL/kg/km)/VO_2_‐max (mL/min/kg)·100] during the 10‐km run was calculated. It showed that FVO_2_‐max did not change with the intervention period (Pre: 84.1 ± 1.3% vs. Post: 85.1 ± 1.2%). In agreement, Iaia et al. (2009) observed a FVO_2_‐max of 84.8% and 81.6% at v10‐km (14.5 km/h) before and after, respectively, a 4‐wk intervention period with speed endurance training. And in the study by Bangsbo et al. (2009), FVO_2_‐max was 85.7% and 83.6% at v10‐km (16.0 km/h) before and after, respectively, a 6–9‐week period with speed endurance training and a basic volume of aerobic training in trained runners. These observations suggest that changes in FVO_2_‐max do not explain the improved 10‐km performance with speed endurance training and reduced training volume. Thus, the improved performance of the 10‐km run appears mainly to be caused by the better running economy. It should be noted, however, that the anaerobic energy production during the 10‐km run, which is suggested to amount up to 20% of the energy provided during a 10‐km run (Joyner and Coyle 2008), is not taken into account in the calculation. In the present study, anaerobic energy production may have been higher after the speed endurance training period due to a possible higher anaerobic capacity reflected by the finding of unchanged VO_2_‐max and higher maximal speed during the incremental test. In support, maximal activity of PFK was higher after the intervention period, which theoretically may have promoted a higher energy production from glycolysis during the 10‐km run.

In summary, running economy was improved after 40 days of intense and reduced volume of training, which may have been related to a reduced expression of UCP3 and higher expression of dystrophin in ST muscle fibers and lower expression of SERCA1 in FT muscle fibers. The finding that running economy at 60% VO_2_‐max in a ST muscle fiber glycogen‐depleted condition was unchanged, suggests that the change in running economy was due to adaptation in ST muscle fibers. The better running economy may explain the improved 10‐km running performance together with a possibly higher anaerobic capacity.

## Conflict of Interest

None declared.

## References

[phy213601-bib-0001] Bangsbo, J. 2015 Performance in sports ‐ with specific emphasis on the effect of intensified training. Scand. J. Med. Sci. Sport 25:88–99. 10.1111/sms.12605 26589122

[phy213601-bib-0002] Bangsbo, J. , T. P. Gunnarsson , J. Wendell , L. Nybo , and M. Thomassen . 2009 Reduced volume and increased training intensity elevate muscle Na+‐K+ pump 2‐subunit expression as well as short‐ and long‐term work capacity in humans. J. Appl. Physiol. 107:1771–1780. 19797693 10.1152/japplphysiol.00358.2009

[phy213601-bib-0003] Barclay, C. J. , J. K. Constable , and C. L. Gibbs . 1993 Energetics of fast‐ and slow‐twitch muscles of the mouse. J. Physiol. 472:61–80. 8145164 10.1113/jphysiol.1993.sp019937 PMC1160477

[phy213601-bib-0004] Berchtold, M. W. , H. Brinkmeier , and M. Müntener . 2000 Calcium ion in skeletal muscle: its crucial role for muscle function, plasticity, and disease. Physiol. Rev. 80:1215–1265. 10893434 10.1152/physrev.2000.80.3.1215

[phy213601-bib-0005] Bergstrom, J. 1962 Muscle electrolytes in man. Scand. J. Clin. Lab. Investig. 68:1–110.

[phy213601-bib-0006] Bickham, D. C. , D. J. Bentley , P. F. Le Rossignol , and D. Cameron‐Smith . 2006 The effects of short‐term sprint training on MCT expression in moderately endurance‐trained runners. Eur. J. Appl. Physiol. 96:636–643. 16408234 10.1007/s00421-005-0100-x

[phy213601-bib-0007] Boss, O. , T. Hagen , and B. B. Lowell . 2000 Uncoupling proteins 2 and 3: potential regulators of mitochondrial energy metabolism. Diabetes 49:143–156. 10868929 10.2337/diabetes.49.2.143

[phy213601-bib-0008] Chopard, A. , N. Arrighi , A. Carnino , and J. F. Marini . 2005 Changes in dysferlin, proteins from dystrophin glycoprotein complex, costameres, and cytoskeleton in human soleus and vastus lateralis muscles after a long‐term bedrest with or without exercise. FASEB J. 19:1722–1724. 16046473 10.1096/fj.04-3336fje

[phy213601-bib-0009] Christensen, P. M. , T. P. Gunnarsson , M. Thomassen , D. P. Wilkerson , J. J. Nielsen , and J. Bangsbo . 2015 Unchanged content of oxidative enzymes in fast‐twitch muscle fibers and kinetics after intensified training in trained cyclists. Physiol. Rep. 3:e12428. 26152692 10.14814/phy2.12428 PMC4552518

[phy213601-bib-0010] Clausen, T. , C. Van Hardeveld , M. E. Everts , H. C. Van , and M. E. Everts . 1991 Significance of cation transport in control of energy metabolism and thermogenesis. Physiol. Rev. 71:733–774. 2057526 10.1152/physrev.1991.71.3.733

[phy213601-bib-0011] Crow, M. T. , and M. J. Kushmerick . 1982 Chemical energetics of slow‐ and fast‐twitch muscles of the mouse. J. Gen. Physiol. 79:147–166. 7061985 10.1085/jgp.79.1.147 PMC2215489

[phy213601-bib-0012] Delbono, O. , and G. Meissner . 1996 Sarcoplasmic reticulum Ca2+ release in rat slow‐ and fast‐twitch muscles. J. Membr. Biol. 151:123–130. 8661500 10.1007/s002329900063

[phy213601-bib-0013] Egan, B. , and J. R. Zierath . 2013 Exercise metabolism and the molecular regulation of skeletal muscle adaptation. Cell Metab. 17:162–184. 23395166 10.1016/j.cmet.2012.12.012

[phy213601-bib-0014] Egan, B. , B. P. Carson , P. M. Garcia‐Roves , A. V. Chibalin , F. M. Sarsfield , N. Barron , et al. 2010 Exercise intensity‐dependent regulation of peroxisome proliferator‐activated receptor coactivator‐1 mRNA abundance is associated with differential activation of upstream signalling kinases in human skeletal muscle. J. Physiol. 588:1779–1790. 20308248 10.1113/jphysiol.2010.188011 PMC2887994

[phy213601-bib-0015] Froemming, G. R. , B. E. Murray , S. Harmon , D. Pette , and K. Ohlendieck . 2000 Comparative analysis of the isoform expression pattern of Ca2+‐regulatory membrane proteins in fast‐twitch, slow‐twitch, cardiac, neonatal and chronic low‐frequency stimulated muscle fibers. Biochim. Biophys. Acta – Biomembr. 1466:151–168. 10.1016/s0005-2736(00)00195-4 10825439

[phy213601-bib-0016] Gollnick, P. D. , K. Piehl , and B. Saltin . 1974 Selective glycogen depletion pattern in human muscle fibres after exercise of varying intensity and at varying pedalling rates. J. Physiol. 241:45–57. 4278539 10.1113/jphysiol.1974.sp010639 PMC1331071

[phy213601-bib-0017] Gong, D. W. , Y. He , M. Karas , and M. Reitman . 1997 Uncoupling protein‐3 is a mediator of thermogenesis regulated by thyroid hormone, b3‐adrenergic agonists, and leptin. J. Biol. Chem. 272:24129–24132. 9305858 10.1074/jbc.272.39.24129

[phy213601-bib-0018] Green, H. J. , M. Burnett , H. Kollias , J. Ouyang , I. Smith , and S. Tupling . 2011 Malleability of human skeletal muscle sarcoplasmic reticulum to short‐term training. Appl. Physiol. Nutr. Metab. 36:904–912. 22087796 10.1139/h11-114

[phy213601-bib-0019] Gunnarsson, T. P. , P. M. Christensen , K. Holse , D. Christiansen , and J. Bangsbo . 2012 Effect of additional speed endurance training on performance and muscle adaptations. Med. Sci. Sports Exerc. 44:1942–1948. 22617392 10.1249/MSS.0b013e31825ca446

[phy213601-bib-0020] He, Z. H. , R. Bottinelli , M. A. Pellegrino , M. A. Ferenczi , and C. Reggiani . 2000 ATP consumption and efficiency of human single muscle fibers with different myosin isoform composition. Biophys. J . 79:945–961. 10920025 10.1016/S0006-3495(00)76349-1 PMC1300991

[phy213601-bib-0021] Howley, E. T. , D. R. Bassett , and H. G. Welch . 1995 Criteria for maximal oxygen uptake: review and commentary. Med. Sci. Sports Exerc. 27:1292–1301. 8531628

[phy213601-bib-0022] Hughes, D. C. , M. A. Wallace , and K. Baar . 2015 Effects of aging, exercise, and disease on force transfer in skeletal muscle. Am. J. Physiol. – Endocrinol. Metab. 309:E1–E10. 25968577 10.1152/ajpendo.00095.2015 PMC4490334

[phy213601-bib-0023] Iaia, F. M. , and J. Bangsbo . 2010 Speed endurance training is a powerful stimulus for physiological adaptations and performance improvements of athletes. Scand. J. Med. Sci. Sport 20:11–23. 10.1111/j.1600-0838.2010.01193.x 20840558

[phy213601-bib-0024] Iaia, F. M. , M. Thomassen , H. Kolding , T. Gunnarsson , J. Wendell , T. Rostgaard , et al. 2008 Reduced volume but increased training intensity elevates muscle Na+‐K+ pump alpha1‐subunit and NHE1 expression as well as short‐term work capacity in humans. J. Appl. Physiol. 106:73–80. 18094063 10.1152/ajpregu.00666.2007

[phy213601-bib-0025] Iaia, F. M. , Y. Hellsten , J. J. Nielsen , M. Fernstrom , K. Sahlin , J. Bangsbo , et al. 2009 Four weeks of speed endurance training reduces energy expenditure during exercise and maintains muscle oxidative capacity despite a reduction in training volume. J. Appl. Physiol. 106:73–80. 18845781 10.1152/japplphysiol.90676.2008

[phy213601-bib-0026] Jansson, E. , and L. Kaijser . 1977 Muscle adaptation to extreme endurance training in man. Acta Physiol. Scand. 100:315–324. 144412 10.1111/j.1748-1716.1977.tb05956.x

[phy213601-bib-0027] Joyner, M. J. , and E. F. Coyle . 2008 Endurance exercise performance: the physiology of champions. J. Physiol. 586:35–44. 17901124 10.1113/jphysiol.2007.143834 PMC2375555

[phy213601-bib-0028] Krustrup, P. , K. Soderlund , M. Mohr , and J. Bangsbo . 2004 Slow‐twitch fiber glycogen depletion elevates moderate‐exercise fast‐twitch fiber activity and O_2_ uptake. Med. Sci. Sports Exerc. 36:973–982. 15179167 10.1249/01.mss.0000128246.20242.8b

[phy213601-bib-0029] Krustrup, P. , N. H. Secher , M. U. Relu , Y. Hellsten , K. Soderlund , and J. Bangsbo . 2008 Neuromuscular blockade of slow twitch muscle fibres elevates muscle oxygen uptake and energy turnover during submaximal exercise in humans. J. Physiol. 586:6037–6048. 18955384 10.1113/jphysiol.2008.158162 PMC2655428

[phy213601-bib-0030] Lowry, O. H. , and J. V. Passonneau . 1972 A flexible system of enzymatic analysis. P. 237 Academic, New York.

[phy213601-bib-0031] Majerczak, J. , J. Karasinski , and J. A. Zoladz . 2008 Training induced decrease in oxygen cost of cycling is accompanied by down‐regulation of serca expression in human vastus lateralis muscle. J. Physiol. Pharmacol. 59:589–602. 18953100

[phy213601-bib-0032] Majerczak, J. , M. Korostynski , Z. Nieckarz , Z. Szkutnik , K. Duda , and J. A. Zoladz . 2012 Endurance training decreases the non‐linearity in the oxygen uptake‐power output relationship in humans. Exp. Physiol. 97:386–399. 22198015 10.1113/expphysiol.2011.062992

[phy213601-bib-0033] Mogensen, M. , M. Bagger , P. K. Pedersen , M. Fernström , and K. Sahlin . 2006 Cycling efficiency in humans is related to low UCP3 content and to type I fibres but not to mitochondrial efficiency. J. Physiol. 571:669–681. 16423857 10.1113/jphysiol.2005.101691 PMC1805795

[phy213601-bib-0340] Murphy, R. M. 2011 Enhanced technique to measure proteins in single segments of human skeletal muscle fibers: fiber‐type dependence of AMPK‐α1 and ‐β1. J Appl Physiol. 110:820–825. http://doi.org/10.1152/japplphysiol.01082.2010 21088205 10.1152/japplphysiol.01082.2010

[phy213601-bib-0034] Nordsborg, N. B. , C. Lundby , L. Leick , and H. Pilegaard . 2010 Relative workload determines exercise‐induced increases in PGC‐1*α* mRNA. Med. Sci. Sports Exerc. 42:1477–1484. 20139785 10.1249/MSS.0b013e3181d2d21c

[phy213601-bib-0035] Prins, K. W. , J. L. Humston , A. Mehta , V. Tate , E. Ralston , and J. M. Ervasti . 2009 Dystrophin is a microtubule‐associated protein. J. Cell Biol. 186:363–369. 19651889 10.1083/jcb.200905048 PMC2728405

[phy213601-bib-0036] Russell, A. P. , E. Somm , M. Praz , A. Crettenand , O. Hartley , A. Melotti , et al. 2003a UCP3 protein regulation in human skeletal muscle fibre types I, IIa and IIx is dependent on exercise intensity. J. Physiol. 550:855–861. 12794174 10.1113/jphysiol.2003.040162 PMC2343085

[phy213601-bib-0037] Russell, A. P. , G. Wadley , M. K. C. Hesselink , G. Schaart , S. Lo , B. Léger , et al. 2003b UCP3 protein expression is lower in type I, IIa and IIx muscle fiber types of endurance‐trained compared to untrained subjects. Pflugers Arch. Eur. J. Physiol. 445:563–569. 12634927 10.1007/s00424-002-0943-5

[phy213601-bib-0038] Rybakova, I. N. , J. R. Patel , and J. M. Ervasti . 2000 The dystrophin complex forms a mechanically strong link between the sarcolemma and costameric actin. J. Cell Biol. 150:1209–1214. 10974007 10.1083/jcb.150.5.1209 PMC2175263

[phy213601-bib-0039] Saunders, P. U. , D. B. Pyne , R. D. Telford , and J. A. Hawley . 2004 Factors affecting running economy in trained distance runners. Sport Med. 34:465–485. 10.2165/00007256-200434070-00005 15233599

[phy213601-bib-0040] Schiaffino, S. , and C. Reggiani . 2011 Fiber types in mammalian skeletal muscles. Physiol. Rev. 91:1447–1531. 22013216 10.1152/physrev.00031.2010

[phy213601-bib-0041] Skovgaard, C. , P. M. Christensen , S. Larsen , T. Andersen , M. Thomassen , and J. Bangsbo . 2014 Concurrent speed endurance and resistance training improves performance, running economy, and muscle NHE1 in moderately trained runners. J. Appl. Physiol. 117:1097–1109. 25190744 10.1152/japplphysiol.01226.2013

[phy213601-bib-0042] Skovgaard, C. , N. Brandt , H. Pilegaard , and J. Bangsbo . 2016 Combined speed endurance and endurance exercise amplify the exercise‐induced PGC‐1*α* and PDK4 mRNA response in trained human muscle. Physiol. Rep. 4:e12864. 27456910 10.14814/phy2.12864 PMC4962071

[phy213601-bib-0043] Smith, I. C. , E. Bombardier , C. Vigna , and A. R. Tupling . 2013 ATP consumption by sarcoplasmic reticulum Ca2+ pumps accounts for 40–50% of resting metabolic rate in mouse fast and slow twitch skeletal muscle. PLoS ONE 8:1–11. 10.1371/journal.pone.0068924 PMC3698183 23840903

[phy213601-bib-0044] Vøllestad, N. K. , and P. C. Blom . 1985 Effect of varying exercise intensity on glycogen depletion in human muscle fibres. Acta Physiol. Scand. 125:395–405. 4083044 10.1111/j.1748-1716.1985.tb07735.x

[phy213601-bib-0045] Walsh, B. , R. A. Howlett , C. M. Stary , C. A. Kindig , and M. C. Hogan . 2006 Measurement of activation energy and oxidative phosphorylation onset kinetics in isolated muscle fibers in the absence of cross‐bridge cycling. Am. J. Physiol. Regul. Integr. Comp. Physiol. 290:R1707–R1713. 16424084 10.1152/ajpregu.00687.2005

